# HER2-positive breast cancer with invasive micropapillary carcinoma component shows immunosuppressive microenvironment and resistance to neoadjuvant therapy

**DOI:** 10.3389/fimmu.2025.1623675

**Published:** 2025-08-01

**Authors:** Lulu Zhang, Lijia Zhou, Anli Yang, Shaoquan Zheng, Keming Chen, Yutian Zou, Qingru Zhou, Daining Wang, Fei Xu, Jiajia Huang, Zhongyu Yuan, Shusen Wang, Yanxia Shi, Peng Sun, Xin An

**Affiliations:** ^1^ Department of Medical Oncology, State Key Laboratory of Oncology in South China, Collaborative Innovation Center for Cancer Medicine, Sun Yat-sen University Cancer Center, Guangzhou, China; ^2^ Department of Breast Surgery, State Key Laboratory of Oncology in South China, Collaborative Innovation Center for Cancer Medicine, Sun Yat-sen University Cancer Center, Guangzhou, China; ^3^ Department of Pathology, State Key Laboratory of Oncology in South China, Collaborative Innovation Center for Cancer Medicine, Sun Yat-sen University Cancer Center, Guangzhou, China

**Keywords:** HER-2 positive invasive micropapillary carcinoma, resistance to neoadjuvant therapy, immunosuppressive tumor microenvironment, *CTNNB1*, β-catenin

## Abstract

**Background:**

Invasive micropapillary carcinoma (IMPC) is a rare histopathological subtype of breast cancer (BC) that shows a high incidence of human epidermal growth factor receptor 2 (HER2)-positive expression. However, the therapeutic efficacy of current standard anti-HER2 therapies for this distinct BC subtype remains unclear.

**Methods:**

We retrospectively analyzed patients with HER2-positive BC who underwent neoadjuvant therapy with trastuzumab (H) or trastuzumab plus pertuzumab (HP) between 2015 and 2023 at Sun Yat-sen University Cancer Center. On the basis of the presence of an IMPC component in pretreatment tumor samples, patients were stratified into IMPC and non-IMPC groups. Baseline clinical and pathological characteristics, pathological complete response (pCR) rates, and survival outcomes were compared between two groups. Additionally, gene expression profiles and immune cells infiltration were assessed using GSE66418 dataset obtained from the Gene Expression Omnibus and ImmuCellAI databases. To validate bioinformatics findings, matched pretreatment tumor samples from both groups were analyzed.

**Results:**

Among the 244 patients included in the study, 38 had an IMPC component (IMPC group), whereas 206 did not (non-IMPC group). The IMPC group exhibited significantly lower pCR rates compared to the non-IMPC group: 21.6% vs. 47.1% (*P* = 0.004) overall, 15.0% vs. 28.4% (*P* = 0.223) in the H-based subgroup, and 27.8% vs. 57.6% (*P* = 0.017) in the HP-based subgroup. IMPC patients also showed worse disease-free survival (*P* < 0.001) and overall survival (*P* = 0.0482) than non-IMPC patients. Bioinformatics analysis revealed that the *CTNNB1* gene, which encodes the β-catenin protein, was the most highly upregulated gene in IMPC patients. Immune profiling demonstrated reduced infiltration of CD4^+^ and CD8^+^ T cells, along with increased macrophage levels in the IMPC tumor microenvironment (TME). Further validation using matched tumor samples confirmed decreased levels of tumor-infiltrating lymphocytes, CD4^+^ and CD8^+^ T cells, elevated M2 macrophages, and higher programmed death-ligand 1 (PD-L1) expression in the IMPC group.

**Conclusion:**

HER2-positive BC with IMPC demonstrates intrinsic resistance to anti-HER2 neoadjuvant therapy and harbors an immunosuppressive TME. These findings highlight the need for alternative treatment strategies and warrant prospective validation.

## Introduction

Invasive micropapillary carcinoma (IMPC) is a rare but clinically significant subtype of breast cancer (BC) (1). It was first described by Fisher et al. in 1980 and subsequently classified as a distinct histological subtype by the World Health Organization in 2003 (2, 3). This unique variant demonstrates characteristic histomorphological features including eosinophilic cytoplasm, distinctive morula-like cell clusters, and prominent stromal retraction spaces—a peculiar architectural pattern where tumor cells exhibit inverted polarity with apical surfaces oriented toward the stromal interface rather than glandular lumina (4, 5). According to the literature, 2%–8% of BC exhibit a micropapillary histological component (mixed IMPC), whereas pure micropapillary carcinoma is infrequent, accounting for only 0.9%–2% of BC (6). Clinically, IMPC typically presents with more aggressive features, including larger tumor size, frequent lymphovascular invasion (LVI), and higher rates of axillary lymph node (LN) involvement at diagnosis (7, 8). IMPC also demonstrates high rates of hormone receptor (HR) positivity and human epidermal growth factor receptor 2 (HER2) overexpression with reported HER2-positive status ranging from 30% to 50% (9). Despite these well-characterized clinicopathological features, critical gaps remain in our understanding of IMPC’s therapeutic responsiveness. Mercogliano et al. reported that the presence of IMPC component correlated with inferior disease-free survival (DFS) in HER2-positive BC receiving adjuvant trastuzumab (H)–based chemotherapy (10). However, the efficacy of modern anti-HER2 therapies, particularly in the neoadjuvant setting, remains poorly defined. This represents a significant clinical concern as trastuzumab and pertuzumab (HP)–based neoadjuvant therapy has become the standard of care for locally advanced HER2-positive BC (11). Our clinical observations of suboptimal responses to anti-HER2 therapy in IMPC cases, both in neoadjuvant and metastatic settings, prompted this comprehensive investigation. This study aims to (1) systematically evaluate the efficacy of contemporary neoadjuvant regimens in HER2-positive IMPC versus conventional carcinomas, (2) characterize distinct molecular features through bioinformatics analysis of gene expression profiles, and (3) elucidate differences in the tumor immune microenvironment through comparative assessment of pretreatment specimens.

## Methods

### Patients and samples

We conducted a retrospective analysis of HER2-positive BC who received standard H- or HP-based neoadjuvant treatment at Sun Yat-sen University Cancer Center between 2015 and 2023. Clinical data were extracted from the electronic medical records. The histopathological slides were reviewed by an independent pathologist to assess the presence of IMPC component. Pre-neoadjuvant tumor samples containing IMPC component (either pure or mixed with invasive ductal carcinoma) were defined as the IMPC group; otherwise, they were classified as the non-IMPC group. Pretreatment tumor samples from the IMPC and non-IMPC groups were obtained to confirm the bioinformatics findings. This study has been approved by the Ethics Committee of Sun Yat-sen University Cancer Center, and informed consent was obtained from all patients (B2024-418-01).

### Definitions and efficacy evaluation

HR-positive was defined as >1% of tumor cells showing positive staining for either estrogen receptors or progesterone receptors by immunohistochemistry (IHC). HER2-positive was defined as IHC scores 3+ or 2+ with fluorescence *in situ* hybridization–positive. Pathological complete response (pCR) was classified into two categories: total pathological complete response (tpCR) and breast pathological complete response (bpCR). TpCR indicated ypT0ypN0, whereas bpCR allowed the presence of carcinoma *in situ* in the breast and any residual tumor in LNs, that is, ypT0/is ypN0/+. The primary breast tumor’s response was evaluated using the Miller–Payne (MP) grading system. Grade 5 (G5) was equivalent to bpCR. Grades 1–4 (G1–G4) indicated varying degrees of residual invasive cancer (non-pCR). DFS was defined as the time from pathological diagnosis to either disease recurrence or death from any cause, whichever occurs first. Overall survival (OS) was defined as the time from pathological diagnosis to death from any cause.

### Bioinformatics analysis to identify hub gene and immune cell infiltration

GSE66418 dataset from the Gene Expression Omnibus database that includes gene expression data from 73 IMPC and 51 non-IMPC samples was downloaded and performed using the Affymetrix Human Genome U133 Plus 2.0 Array. R software (v4.0.4) was used to normalize the GSE66418 dataset’s expression matrix. Differentially expressed genes (DEGs) were identified using the “limma” R package with criteria of adjusted *P*-value < 0.05 and |logFC| ≥ 1. Enrichment analysis for Kyoto Encyclopedia of Genes and Genomes (KEGG) and Gene Ontology (GO) was performed using “ClusterProfiler” with a significance threshold of corrected *P*-value < 0.05. The protein-protein interaction (PPI) network for *Homo sapiens* was built using the STRING database with a minimum confidence score of 0.4. It was visualized and analyzed in Cytoscape (v3.x), where hub genes were identified using the CytoHubba plugin with the maximal clique centrality (MCC) algorithm. Immune cell abundance was assessed by ImmuCellAI, which includes 18 T-cell subtypes and six other immune cells (B cells, natural killer cells, monocytes, macrophages, neutrophils, and dendritic cells).

### IHC and TIL assessment

Tissue sections were deparaffinized, hydrated, and stained with specific antibodies. The slides were then scanned at ×100 magnification to produce high-resolution digital images for analysis. The image data were uploaded to the HALO Pathology Image Analysis system for analysis using the Multiplex IHC (v3.1.1) algorithm, which objectively quantified total and positively stained cell counts in the specified area, ensuring accurate assessment of protein expression levels across samples. The evaluation of tumor-infiltrating lymphocytes (TILs) was performed according to a standardized five-step scoring system developed by the International Immuno-oncology Biomarker Working Group. TILs include all mononuclear cells (lymphocytes and plasma cells) while excluding neutrophils. The percentage of stromal TILs within the invasive tumor borders was assessed, excluding areas with crush artifacts, necrosis, or regressive hyalinization.

### Statistical analyses

Statistical analyses were conducted with SPSS v24.0 and R v4.0.4. Descriptive statistics summarized clinicopathological characteristics, and the χ²- test evaluated baseline clinical differences and primary breast tumor’s response to neoadjuvant therapy between two groups. Spearman’s test analyzed the correlation between immune infiltration and hub genes, illustrated with a scatter diagram. Patients in the IMPC and non-IMPC groups were matched 1:1 using propensity score matching according to age, tumor stage, HR status, histological grade, Ki-67 status, and neoadjuvant regimens. The nearest neighbor method with a caliper of 0.2 was applied in R to minimize baseline differences. Survival analysis was performed using Kaplan–Meier survival curves, and group differences were compared with the log-rank test. Protein expression levels in pre-neoadjuvant tumor samples were compared between groups using Wilcoxon non-parametric tests. A *P-*value < 0.05 was considered statistically significant.

## Results

### Baseline characteristics

**Figure 1:**
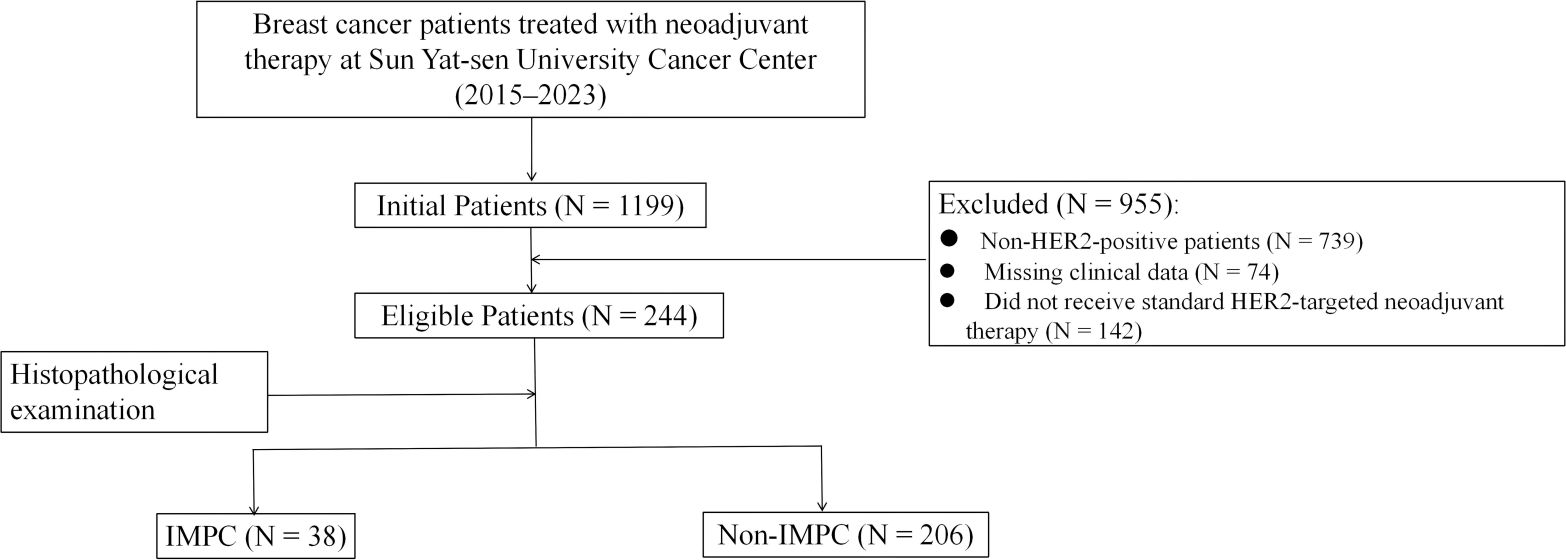
Flow diagram of patients’ selection.

**Figure 2:**
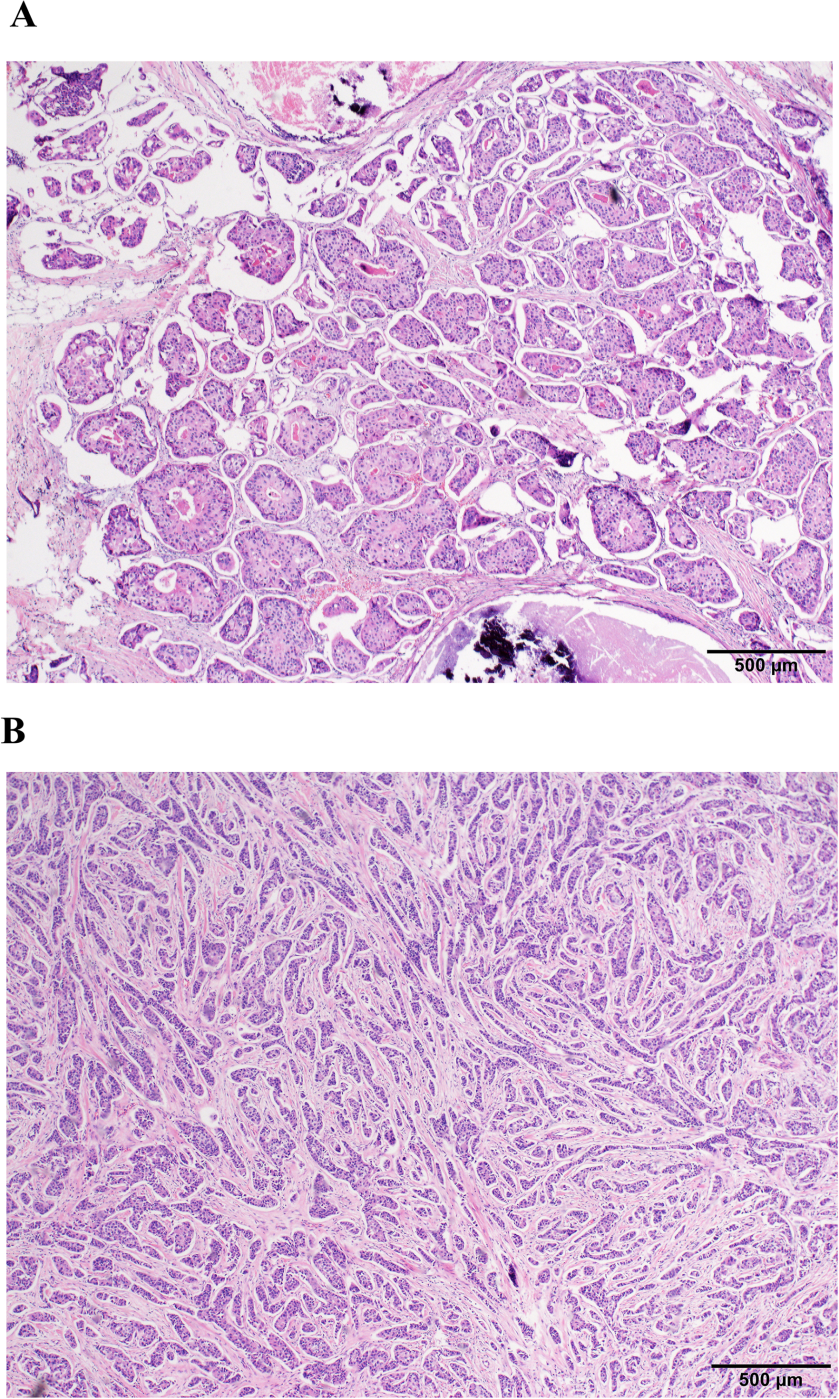
Histopathological comparison of IMPC and non-IMPC (H&E stain; scale bar, 500 µm). **(A)** IMPC morphology. **(B)** Non-IMPC morphology.

The patient selection process is detailed in **Figure 1**. Our final cohort comprised 244 patients who completed standard neoadjuvant therapy followed by surgical resection and had complete clinicopathological data available for analysis. Neoadjuvant regimens consisted of six cycles of docetaxel and carboplatin with H or HP (TCbH ± P) or four cycles of anthracyclines and cyclophosphamide followed by four cycles docetaxel with H or HP (AC-TH ± P). The study identified 38 (15.6%) cases, including 10 cases with pure IMPC and 28 cases with mixed IMPC (10%–90% IMPC component) (**Figure 2**). Patients in the IMPC group showed a higher percentage of HR-positive expression compared with patients in the non-IMPC group (71.1% vs. 61.7%, *P* < 0.05). Patients in the IMPC group also showed a trend of high Ki-67 index and younger age, although the differences showed no significance (**Table 1**).

**Table 1:** Baseline characteristics.

Characteristic	Total (n = 244)	IMPC (n = 38)	Non-IMPC (n = 206)	*^a^P*-value
Median age (year)	46 (25–74)	44 (25–66)	47 (25–74)	0.133
Age, n (%)				0.183
≤40	80 (32.8)	16 (42.1)	64 (31.1)	
>40	164 (67.2)	22 (57.9)	142 (68.9)	
Tumor size, n (%)				0.748
≤ 2 cm	20 (8.2)	2 (5.3)	18 (8.7)	
> 2 cm	224 (91.8)	36 (94.7)	188 (91.3)	
Lymph nodes, n (%)				0.406
Negative	12 (4.9)	3 (7.9)	9 (4.4)	
Positive	232 (95.1)	35 (92.1)	197 (95.6)	
Hormone receptor, n (%)				<0.05
Negative	90 (36.9)	11 (28.9)	79 (38.3)	
Positive	154 (63.1)	27 (71.1)	127 (61.7)	
Grade, n (%)				0.993
G1 + G2	135 (55.3)	21 (55.3)	114 (55.3)	
G3	109 (44.7)	17 (44.7)	92 (44.7)	
Ki-67, n (%)				0.254
≤30%	104 (42.6)	13 (34.2)	91 (44.2)	
>30%	140 (57.4)	25 (65.8)	115 (55.8)	
Neoadjuvant targeted therapy, n (%)				0.052
Trastuzumab	94 (38.5)	20 (52.6)	74 (35.9)	
Trastuzumab + pertuzumab	150 (61.5)	18 (47.4)	132 (64.1)	
Neoadjuvant chemotherapy, n (%)				
AC-T	170 (69.7)	24 (63.2)	146 (70.9)	0.342
TCb	74 (30.3)	14 (35.8)	60 (29.1)	

^a^ *P*-values were calculated with the use of the chi-square test between IMPC group and non-IMPC group.

Bold values indicate statistically significant differences (*P* < 0.05).

### Patients with IMPC component showed poor response to neoadjuvant treatment

**Figure 3:**
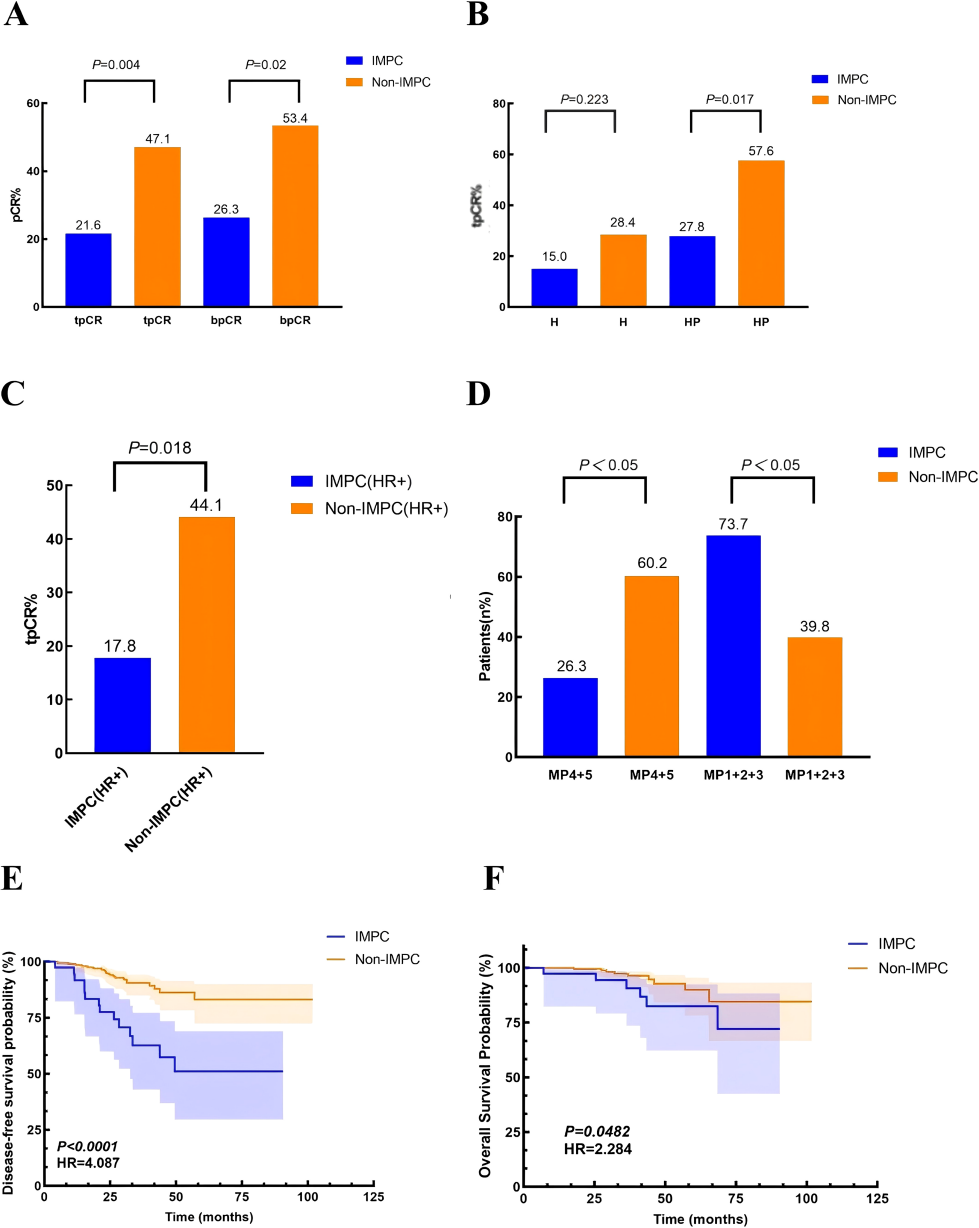
Treatment responses and survival outcomes in IMPC vs. non-IMPC cohorts. **(A)** Comparison of tpCR and bpCR rates between the IMPC and non-IMPC groups. **(B)** The tpCR rates stratified by neoadjuvant trastuzumab **(H)**– or trastuzumab and pertuzumab (HP)–based regimens. **(C)** The tpCR rates in hormone receptor-positive (HR+) subgroups comparing IMPC and non-IMPC patients. **(D)** Neoadjuvant treatment response assessed by the Miller–Payne (MP) grading system for primary breast tumors in the IMPC and non-IMPC groups. **(E, F)** Kaplan–Meier curves demonstrating **(E)** disease-free survival (DFS) and **(F)** overall survival (OS) differences between the IMPC and non-IMPC groups.

Overall, the tpCR rates in the IMPC and non-IMPC groups were 21.6% vs. 47.1% (*P* = 0.004), and bpCR rates were 26.3% vs. 53.4% (*P* = 0.02), respectively (**Figure 3A**). For patients who received H-based neoadjuvant treatment, tpCR rates in the IMPC and non-IMPC groups were 15.0% vs. 28.38% (*P* = 0.223). For patients who received HP-based neoadjuvant treatment, tpCR rates in the IMPC and non-IMPC groups were 27.78% vs. 57.58% (*P* = 0.017) (**Figure 3B**). To exclude the potential impact of HR expression on treatment response, we further compared the tpCR rates between the HR+ IMPC group and the HR+ non-IMPC group. As a result, the IMPC group also demonstrated significant lower tpCR rate than that in the non-IMPC group: 17.8% vs. 44.1%, *P* = 0.018 (**Figure 3C**). Consistently, fewer patients in the IMPC group achieved MP grade 4/5 compared to those in the non-IMPC group: 26.3% vs. 60.2%, *P* < 0.05 (**Figure 3D**). The median follow-up time was 38.7 (range: 7.0–90.5) months for IMPC patients and 37.0 (range: 5.0–101.9) months for non-IMPC patients. Patients in the IMPC group also showed a worse DFS (*P* < 0.001, HR = 4.087) and OS (*P* = 0.0482, HR = 2.284), and 5-year DFS in the IMPC and non-IMPC groups were 51.7% vs. 83.6% (*P* < 0.05) (**Figures 3E, F**).

### IMPC showed a high expression of CTNNB1 gene, β-catenin protein, and immunosuppressive TME

**Figure 4:**
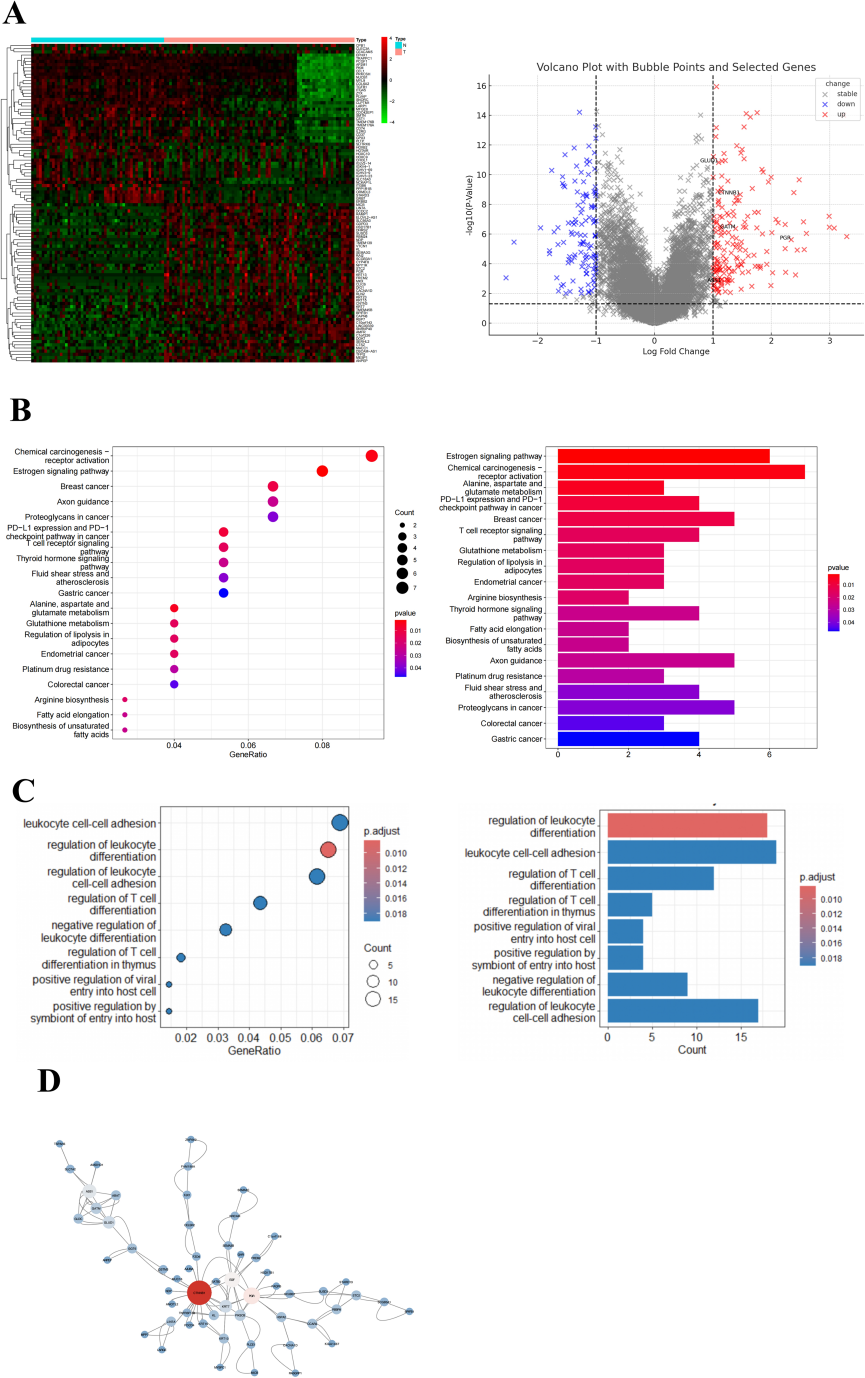
Gene expression and pathway enrichment analysis of DEGs in the GSE66418 dataset. **(A)** Heatmap and volcano plot of DEGs for GSE66418 dataset. Red represents upregulated genes, and blue represents downregulated genes. **(B)** KEGG enrichment results are displayed as bubble plots (left) and bar plots (right). **(C)** GO enrichment results are displayed as bubble plots (left) and bar plots (right). **(D)** Hub gene analysis in the PPI network. The hub genes were identified using the MCC algorithm, highlighting key regulatory genes within the network. Larger node sizes represent genes with higher MCC scores, indicating greater centrality and potential significance in the regulatory network.

**Figure 5:**
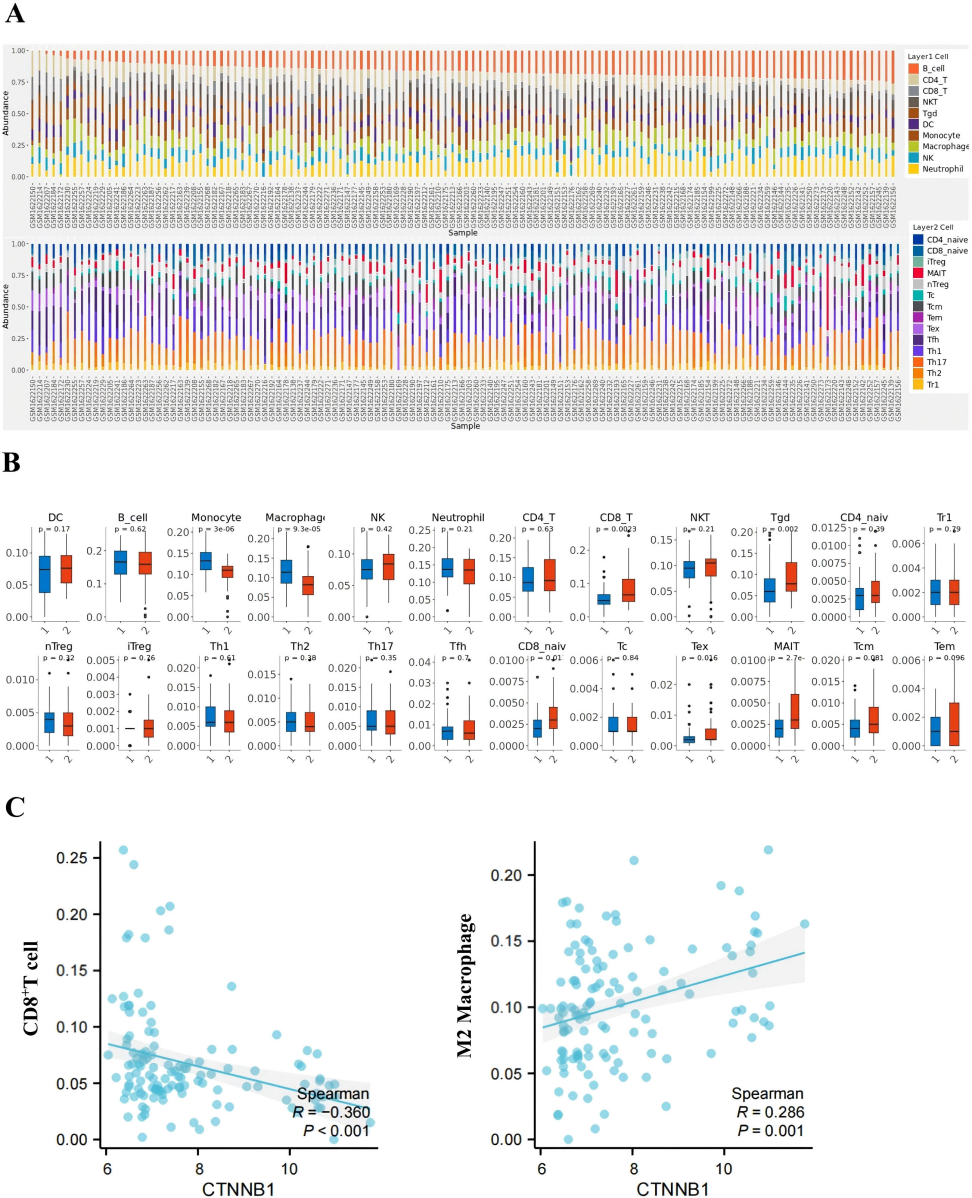
Immune microenvironment profiling in IMPC vs. non-IMPC cohorts. **(A)** Immune cell composition analysis: Stacked bar plot displaying immune cell proportions predicted by ImmuCellAI using transcriptomic data (GSE66418 dataset). Each x-axis bar represents an individual sample (IMPC vs. non-IMPC cohorts). **(B)** Comparative immune cell infiltration: Box plots showing differentially abundant immune cell subsets between IMPC (group 1) and non-IMPC (group 2) samples (*p < 0.05, **p < 0.01, and ***p < 0.001; two-tailed Mann–Whitney U-test). **(C)** *CTNNB1*-immune interaction network: Spearman correlation analysis evaluated the relationship between *CTNNB1* expression level and immune cell infiltration.

**Figure 6:**
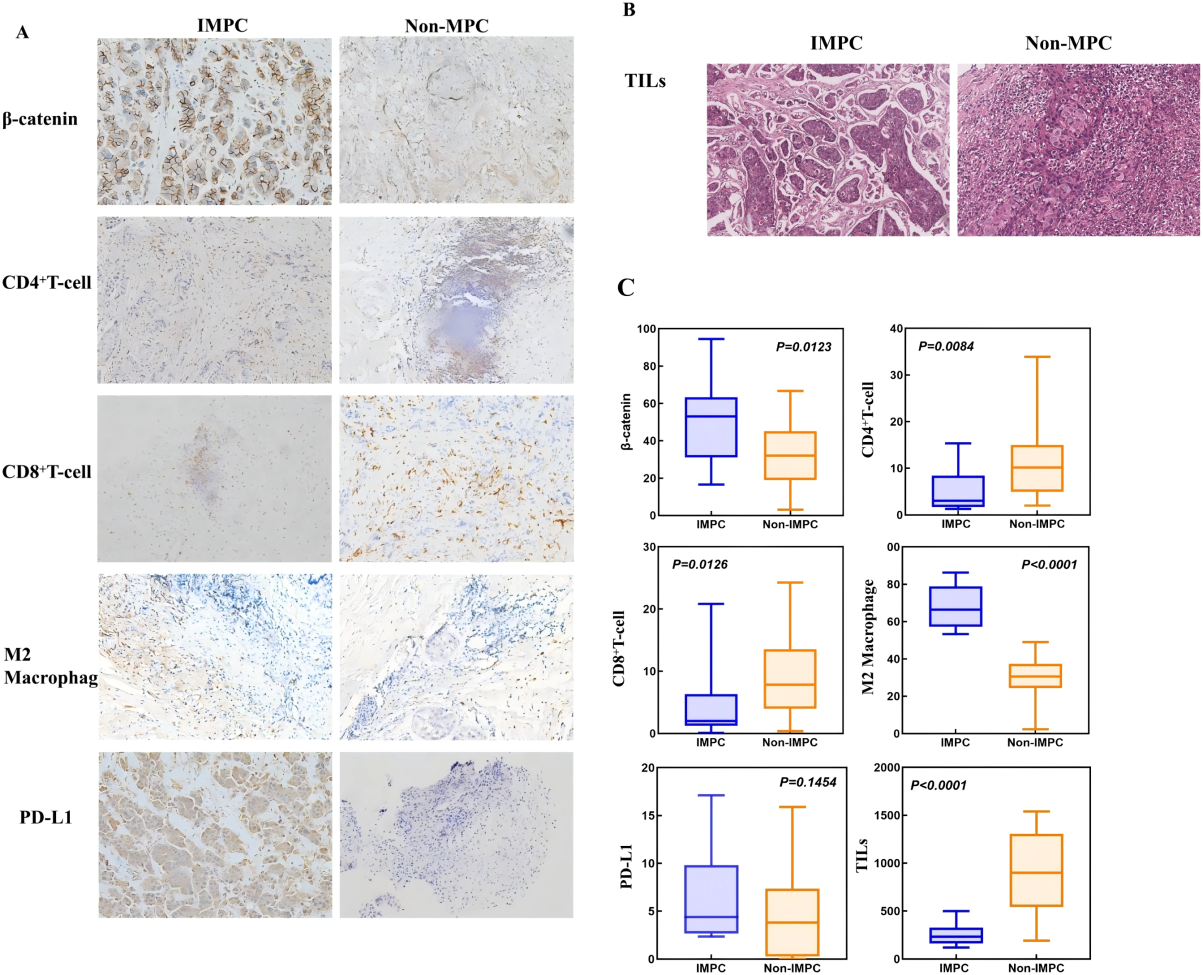
Distinct immune microenvironment features in matched pretreatment IMPC vs. non-IMPC tumor samples. **(A)**The expression of β-catenin, CD8^+^ T cells, CD4^+^ T cells, and M2 macrophages in IMPC and non-IMPC tissues (IHC stain, 200 µm). **(B)** The expression of TILs in IMPC and non-IMPC tissues (H&E stain; scale bar, 200 µm). **(C)** Boxplots showing the expression levels of β-catenin and key immune cells.

Analysis of the GSE66418 dataset identified 300 DEGs between the IMPC and non-IMPC groups, with 181 genes upregulated and 119 downregulated in IMPC (**Figure 4A**). KEGG enrichment showed that upregulated genes were linked to oncogenesis, estrogen signaling, programmed cell death -1/programmed cell death ligand 1 (PD-1/PD-L1) checkpoints, and T-cell receptor signaling pathways (**Figure 4B**). Additionally, GO analysis highlighted enrichment in pathways related to T-cell activation and function (**Figure 4C**). The MCC algorithm in CytoHubba identified *CTNNB1* as the hub gene (**Figure 4D**). ImmunCellAI analysis found significantly lower infiltration of CD8^+^ and CD4^+^ T-cell levels and higher infiltration of macrophage in the IMPC group compared with those in the non-IMPC group (**Figures 5A, B**). *CTNNB1* expression was positively correlated with CD8^+^ T cells and negatively correlated with macrophages (**Figure 5C**). IHC staining of matched pre-neoadjuvant tumor samples confirmed β-catenin that is encoded by CTNNB1 gene had higher expression in the IMPC group compared to that in the non-IMPC group. Moreover, the number of CD4^+^ T cells, CD8**^+^** T cells, and TILs were significantly lower, whereas M2 macrophages were significantly higher in the IMPC group than those in the non-IMPC group. Expression of PD-L1 was shown to be higher in IMPC samples (**Figure 6**).

## Discussion

This study provides the first clinical evidence that IMPC components predict inferior response to H- and HP-based neoadjuvant therapy, along with worse survival outcomes in HER2-positive IMPC patients. Our findings underscore the critical need for routine IMPC component identification during initial diagnostic workup, as this histopathological feature may necessitate alternative treatment strategies.

To explore more appropriate treatment strategies for IMPC, we investigated the potential resistance mechanisms. Initial bioinformatics analysis identified *CTNNB1* as the most upregulated gene in IMPC. The expression of β-catenin protein encoded by *CTNNB1* was also higher in pre-neoadjuvant IMPC tumor samples than that in the non-IMPC samples. Upregulated β-catenin has been reported in IMPC through Wnt signaling and shown to be related to LVI invasion and LN metastasis (12). Our previous work reported the correlation between Wnt/β-catenin and trastuzumab resistance in BC (13). In addition, β-catenin pathway has been shown to suppress antitumor immune response by impairing immune cell recruitment and reducing T- and B-cell infiltration in a variety of cancers, including colon cancer, lung cancer, and melanoma (14–17). In line with this evidence, a significant decrease of immunoactive CD4^+^ and CD8^+^ T cells and an increase of immunosuppressive M2 macrophages were observed in IMPC both from bioinformatics analysis and pretreatment tumor samples assessment. Additionally, significantly lower stromal TILs were also observed in IMPC tumor samples. Accumulating evidence has established the important role of tumor immune microenvironment on treatment response in HER2-positive BC (18, 19). TILs, the most widely used biomarker for TME, have been established as a robust prognostic and predictive biomarker for HER2-positive BC. Elevated TIL levels consistently correlate with enhanced therapeutic responses to anti-HER2 treatments, including H, HP, and tyrosine kinase inhibitors (20–23). Infiltration of immune-active CD4^+^ and CD8^+^ T cells also has been demonstrated to be linked to better responses to trastuzumab and higher pCR rates in HER2-positive BC (24–27). *In vitro* experiments showed that CD4^+^ T cells could boost trastuzumab effectiveness in HER2-positive BC via immune stimulation (28). Conversely, the immunosuppressive TME portends inferior outcomes with anti-HER2 therapies (29–31). Notably, M2 macrophages drive trastuzumab sensitivity by releasing immunosuppressive cytokines like interleukin-10 (IL-10) and transforming growth factor β (TGF-β) and metabolic reprogramming of tumor cells to create an immune-excluded phenotype (32).

Beyond β-catenin–mediated mechanisms, several established HER2 resistance pathways have been reported, such as activation of the phosphatidylinositol 3-kinase / V-akt murine thymoma viral oncogene homolog / mammalian target of the rapamycin (PI3K/AKT/mTOR) pathway, loss of PTEN expression, and increased expression of HER3 and insulin-like rrowth factor 1 receptor (IGF-1R) (33–35). Interestingly, emerging evidence suggests potential crosstalk between Wnt/β-catenin signaling and these classical pathways. For example, β-catenin activation has been linked to PI3K/AKT activation, potentially amplifying HER2 resistance (36, 37). In IMPC tumors, the concomitant presence of β-catenin–driven immunosuppressive microenvironment and enhanced pro-survival signaling may synergistically contribute to resistance against anti-HER2 therapies. Future mechanistic studies are warranted to delineate these interactions.

All these data suggest that β-catenin activation and immunosuppressive microenvironment might be involved in the resistance of IMPC to the current standard H- and HP-based neoadjuvant treatments. The addition of immunotherapy could be a potential strategy to overcome this resistance. For example, the APTneo Michelangelo study showed that adding atezolizumab to neoadjuvant HP plus chemotherapy led to a 9.9% pCR increase (38). The most recently reported neoHIP study reported neoadjuvant pembrolizumab-THP achieved pCR rate of 67.2%, compared with 48.3% of pCR in THP arm (39). Moreover, neoadjuvant T-DXd is also being evaluated in the ongoing DESTINY-Breast11 trial; hopefully, this most robust anti-HER2 ADC might improve the efficacy of IMPC.

This study is also subject to certain limitations. Firstly, the sample size was relatively small, especially for the IMPC group. Although expanding the dataset or incorporating external cohorts would enhance the robustness of our findings, the rarity of HER2-positive IMPC makes this difficult at present. Prospective studies are being planned to validate these results. Nevertheless, we still demonstrated significantly lower pCR rates in the IMPC group. Secondly, due to the lack of study concerning molecular subtypes of IMPC, IMPC database for bioinformatics analysis was not specific to the HER2 subtype, which might cause some bias. Thirdly, because IMPC could only be diagnosed in pre-chemotherapy sample obtained through core needle biopsy, thus we only had limited paraffin section that was insufficient for DNA and RNA testing. To overcome these challenges, further studies have been initiated aiming to investigate the upstream and downstream regulatory mechanisms of the Wnt/β-catenin signaling pathway and its potential crosstalk with the HER2 signaling axis, as well as the cellular heterogeneity within the IMPC tumor microenvironment to deepen our understanding of the molecular basis of IMPC resistance.

## Conclusion

The presence of IMPC components in HER2-positive BC was associated with poor response and survival outcomes to current standard H- and HP-based neoadjuvant treatment. β-Catenin and its associated tumor immunosuppressive microenvironment might be the potential mechanism. Further prospective studies with large sample size are warranted to validate these findings and to investigate more appropriate treatment strategies.

## Data Availability

The original contributions presented in the study are included in the article/supplementary material. Further inquiries can be directed to the corresponding author.
